# Prognostic values of serum alkaline phosphatase and globulin levels in patients undergoing intravenous thrombolysis

**DOI:** 10.3389/fnmol.2022.932075

**Published:** 2022-07-15

**Authors:** Hong-Jing Zhu, Xin Sun, Zhen-Ni Guo, Yang Qu, Ying-Ying Sun, Hang Jin, Mei-Qi Wang, Bao-Feng Xu, Yi Yang

**Affiliations:** ^1^Department of Neurology, China National Comprehensive Stroke Center, The First Hospital of Jilin University, Changchun, China; ^2^Neuroscience Research Centre, The First Hospital of Jilin University, Changchun, China

**Keywords:** acute ischemic stroke, intravenous thrombolysis, alkaline phosphatase, globulin, clinical outcomes

## Abstract

**Background:**

In previous studies, alkaline phosphatase (ALP) level was a prognostic factor for patients with ischemic stroke, and globulin level was associated with hemorrhagic transformation (HT) after intravenous thrombolysis (IVT). However, the association between these serum biomarkers and prognosis in patients with acute ischemic stroke (AIS) who undergo IVT remains unclear. This study aimed to investigate the characteristics of serum ALP and globulin levels after IVT and to assess the relationship between these serum biomarkers and prognosis.

**Materials and methods:**

This retrospective study used a prospectively collected database. We included patients with AIS who received recombinant tissue plasminogen activator (rt-PA) IVT. Demographic information, vascular risk factors, laboratory test results, and other stroke-related data were collected for analysis. Clinical outcomes included HT and 3-month poor outcome (modified Rankin Scale scores ≥ 2) after IVT. The association of ALP and globulin levels with HT and poor outcome was investigated using multivariate logistic regression analysis. An individualized prediction model based on ALP and globulin levels for functional outcomes was established.

**Results:**

We enrolled 750 patients in this study; 452 patients (60.3%) had poor outcome, and 117 patients (15.6%) had HT after IVT. After adjusting for all confounders, serum globulin level [OR = 1.055; 95% confidence intervals (CI): 1.006–1.107; *P* = 0.028] was independently associated with HT in patients with IVT. Serum ALP (OR = 1.009; 95% CI: 1.002–1.016; *P* = 0.010) and globulin levels (OR = 1.062; 95% CI: 1.020–1.107; *P* = 0.004) were associated with 3-month poor outcome in these patients. The constructed individualized prediction model for the 3-month poor outcome comprised the National Institutes of Health Stroke Scale (NIHSS) score, Trial of Org 10172 in Acute Stroke Treatment (TOAST), history of antihypertensive therapy, ALP and globulin levels. The area under the curve of the training and validation sets were 0.726 and 0.706, respectively, revealing that the model had good discriminating power. The *P*-values for the Hosmer-Lemeshow test in the training and validation sets were 0.978 and 0.148, respectively, indicating the model had good calibration.

**Conclusion:**

This study found that higher serum globulin levels were independently associated with HT. Additionally, higher serum ALP and globulin levels were independently associated with a poor outcome in patients after IVT.

## Introduction

Stroke, especially ischemic stroke, is the leading cause of death and disability worldwide (Meschia and Brott, 2018; Collaborators, 2021). Recombinant tissue plasminogen activator (rt-PA) is an approved intravenous thrombolytic agent used to treat acute ischemic stroke (AIS) (Paul and Candelario-Jalil, 2021). Although rt-PA has been proven effective in alleviating neurological deficits and improving clinical outcomes, about half of the patients receiving rt-PA still have a poor prognosis after intravenous thrombolysis (IVT) or complications such as hemorrhagic transformation (HT) (Yaghi et al., 2017). Early determination of risk factors for poor prognosis can help clinicians stratify outcomes and more aggressively determine appropriate treatment for them. Therefore, it is necessary to identify reliable serum biomarkers that can predict the prognosis and HT in patients after IVT.

Alkaline phosphatase (ALP) and globulin are indices of liver function in routine clinical examinations. Previous studies have found that ALP have a crucial role in vascular calcification and might contribute to the acute inflammatory response (Haarhaus et al., 2017). ALP has been evaluated as a potential biomarker for many diseases, including many co-morbidities associated with stroke (Brichacek and Brown, 2019). ALP level was associated with increased adverse events in cardiovascular disease (Park et al., 2013). Recently, elevated ALP levels have been found to be independent risk predictors of adverse outcomes after stroke (Ryu et al., 2010; Zong et al., 2018; Brichacek and Brown, 2019). Besides, serum globulin levels have been used to assess the severity of chronic inflammation (Wu et al., 2019). Some studies have found that globulin level was a risk factor for HT after IVT in patients with AIS (Zhong et al., 2021). However, no study has investigated the association between ALP and globulin levels and the long-term clinical outcomes after intravenous thrombolysis. The prognostic validity of ALP and globulin levels in stroke patients treated with intravenous thrombolysis is not well-understood. Thus, this study aimed to evaluate the characteristics of serum ALP and globulin levels in patients undergoing intravenous thrombolysis and assess the relationship between these serum biomarkers and HT and the 3-month prognosis.

## Materials and methods

### Study population

This retrospective study was conducted using a prospectively collected database of consecutive patients with acute ischemic stroke at the First Hospital of Jilin University. Patients (aged > 18 years) diagnosed with AIS who received standard-dose intravenous thrombolysis with rt-PA between April 2015 and December 2020 were included. Patients were excluded if they met the following criteria: (1) final diagnosis with a stroke mimic, (2) received mechanical thrombectomy after IVT, (3) had modified Rankin Scale (mRS) scores > 2 before the onset of the disease, (4) self-reported or diagnosed with severe liver disease, and (5) incomplete laboratory tests or follow-up data.

The AIS diagnosis was based on clinical signs and symptoms and computed tomography (CT) before IVT. CT was performed 24 h after IVT to detect HT.

### Data collection

The following participants’ data were prospectively collected and recorded in a well-established database: (1) demographic information (age and sex), (2) vascular risk factors (cigarette smoking, alcohol consumption, atrial fibrillation, coronary heart disease, hypertension, diabetes mellitus, dyslipidemia, and previous stroke), (3) history of previous medications (antihypertensive therapy, hypoglycemic therapy, and antiplatelet therapy), (4) baseline National Institutes of Health Stroke Scale (NIHSS) score, (5) baseline systolic blood pressure (SBP) and diastolic blood pressure (DBP), (6) baseline blood glucose, (7) onset-to-needle time (ONT), and (8) clinical and follow-up information. Stroke subtypes were categorized according to the Trial of Org 10172 in Acute Stroke Treatment (TOAST) (Adams et al., 1993). The TOAST classification includes five subtypes of ischemic stroke: (1) large-artery atherosclerosis, (2) cardioembolism, (3) small-vessel occlusion, (4) other determined etiology, and (5) undetermined etiology. ALP and globulin levels were collected from fasting blood samples within 24 h of IVT.

### Outcomes

Clinical outcomes included hemorrhagic transformation and a poor outcome 3 months after thrombolysis. Functional outcomes were assessed using the modified Rankin Scale (mRS) 3 months after IVT. A poor outcome was defined as an mRS score of 2–6, while a favorable outcome was an mRS score of 0–1. HT was defined as any visible hemorrhage observed during cranial CT 24 h after thrombolysis.

### Statistical analysis

Normally distributed continuous variables are expressed as mean ± standard deviation, and continuous variables that did not conform to normal distribution are expressed as the median and interquartile range (IQR). Categorical variables are expressed as frequencies and percentages. Differences between groups of continuous variables were tested using the *t*-test or Mann–Whitney U test, according to normality. Differences between categorical variables were determined using the χ2 test. Multifactorial logistic regression analysis was used to test the correlation between indices and clinical outcomes. Four models were developed for the multivariate analysis: Model 1 was adjusted for age and sex; Model 2 was adjusted for Model 1 + vascular risk factors, including cigarette smoking, alcohol consumption, atrial fibrillation, coronary heart disease, hypertension, diabetes mellitus, dyslipidemia, and previous stroke; Model 3 was adjusted for Model 2 + history of previous medications, including antihypertensive therapy, hypoglycemic therapy, and antiplatelet therapy; and Model 4 was adjusted for Model 3 + baseline NIHSS scores, baseline SBP and DBP, baseline blood glucose, ONT, and TOAST. Odds ratios (OR) and 95% confidence intervals (CI) were used to evaluate the risk of poor outcomes and HT.

An individualized prediction model for functional outcomes, based on serum ALP and globulin levels, was developed using multivariate logistic regression analysis. The included patients were randomly assigned to the training and validation sets in a 7:3 ratio. Variables with *P* < 0.05 in the univariate analysis were included in the multivariate logistic regression analysis using a backward selection method. A nomogram was constructed based on the training set. The discrimination of the prediction model was assessed using the receiver operating characteristic (ROC) curve and area under the curve (AUC), and calibration of the prediction model was assessed using the Hosmer-Lemeshow test. Discrimination and calibration were performed for both the training and validation sets. The model was internally validated using all data by performing a 10-fold cross-validation. The *P* < 0.05 was considered statistically significant. Statistical analyses were performed using the Statistical Program for Social Sciences version 23.0 (SPSS, IBM, West Grove, PA, United States), and the nomogram was performed using Stata 15.0.

### Ethics statement

The study was approved by the Ethics Review Committee of the First Hospital of Jilin University. The participants or their direct relatives provided their written informed consent to participate in this study.

## Results

### Characteristics of study participants

**FIGURE 1:**
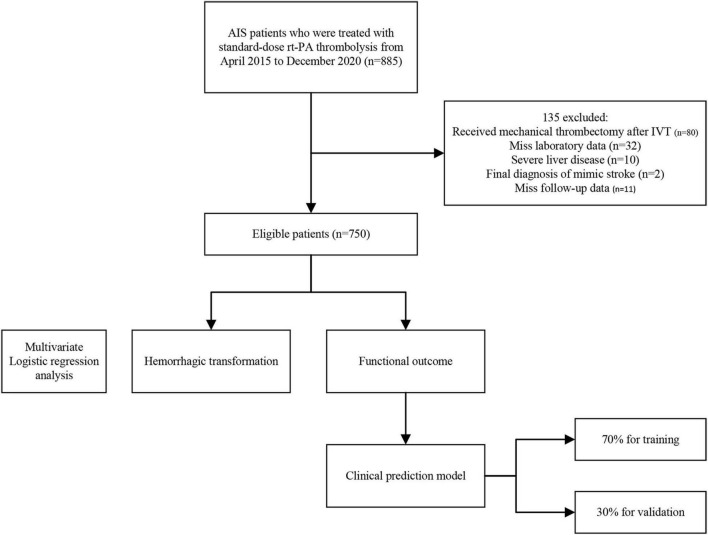
The flowchart of patient selection.

This study included 885 patients; 80 were excluded because they had undergone mechanical thrombectomy after IVT, 55 were excluded because they were finally diagnosed with a stroke mimic (*n* = 2), had severe liver disease (*n* = 10), missed laboratory data (*n* = 32), and follow-up data (*n* = 11). A flowchart of the patient selection process is shown in Figure 1.

Eventually, 750 patients with AIS were enrolled in this study, of whom 543 (72.4%) were male, aged 62 (range: 53–69) years. The median onset-to-needle time was 184 (141–229) minutes, and the median baseline NIHSS score was 8 (5–12). In the study population, 452 patients (60.3%) had poor outcomes (mRS ≥ 2), and 117 patients (15.6%) had hemorrhage transformation after thrombolytic therapy. The baseline clinical characteristics and outcomes are shown in Table 1.

**TABLE 1:** Clinical characteristics of included patients.

Variables	Total (*n* = 750)
**Demographics information**	
Age, years (IQR)	62 (53–69)
Male, *n* (%)	543 (72.4)
**Vascular risk factors**	
Cigarette smoking, *n* (%)	388 (51.7)
Alcohol consumption, *n* (%)	314 (41.9)
Hypertension, *n* (%)	384 (51.2)
Diabetes mellitus, *n* (%)	139 (18.5)
Coronary heart disease, *n* (%)	146 (19.5)
Atrial fibrillation, *n* (%)	74 (9.9)
Previous stroke, *n* (%)	103 (13.7)
Dyslipidemia, *n* (%)	313 (41.7)
**History of previous medications**	
Antihypertensive therapy, *n* (%)	300 (40)
Hypoglycemic therapy, *n* (%)	121 (16.1)
Antiplatelet therapy, *n* (%)	98 (13.1)
Baseline SBP, mmHg (IQR)	154 (139–167)
Baseline DBP, mmHg (IQR)	89 (81–98)
Baseline blood glucose, mmol/L (IQR)	7.6 (6.5–9.2)
Baseline NIHSS score (IQR)	8 (5–12)
ONT, min (IQR)	184 (141–229)
**TOAST**	
Large-artery atherosclerosis, *n* (%)	274 (36.5)
Small-vessel occlusion, *n* (%)	280 (37.3)
Cardioembolism, *n* (%)	84 (11.2)
Other determined etiology, *n* (%)	12 (1.6)
Undetermined etiology, *n* (%)	100 (13.3)
**Laboratory tests**	
ALP, U/L (IQR)	75.7 (62.2–92)
Globulin, g/L (IQR)	27.1 (24.3–29.8)
**Clinical outcomes**	
HT, *n* (%)	117 (15.6)
Poor outcome, *n* (%)	452 (60.3)

IQR, inter quartile range; SBP, systolic blood pressure; DBP, diastolic blood pressure; NIHSS, National Institutes of Health Stroke Scale; ONT, onset to needle time; TOAST, Trial of Org 10172 in acute stroke treatment; ALP, alkaline phosphatase; HT, hemorrhagic transformation.

### The association of alkaline phosphatase and globulin levels with hemorrhagic transformation after intravenous thrombolysis

**FIGURE 2:**
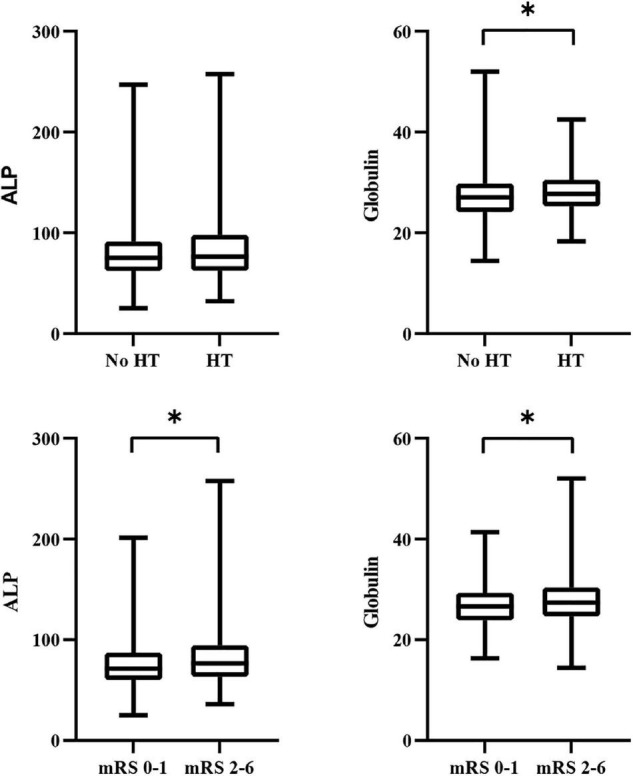
Comparison of the distribution of ALP and globulin levels in clinical outcomes. ALP, alkaline phosphatase; HT, hemorrhagic transformation; mRS, modified Rankin scale. *P < 0.05.

We divided the patients into two groups according to the presence or absence of HT. As shown in Table 2, the distribution in the HT and no-HT groups showed statistically significant differences in atrial fibrillation, baseline SBP, baseline blood glucose, baseline NIHSS score, antiplatelet therapy, TOAST, and globulin levels (*P* < 0.05). We found that globulin levels were higher in patients with HT (27.0 vs. 27.7, *P* = 0.022). However, ALP levels were not significantly different between the HT and no-HT groups (75.1 vs. 76.3, *P* = 0.617). A comparison of the distribution of ALP and globulin levels in HT patients is presented in Figure 2.

**TABLE 2:** Clinical characteristics of patients in the presence/absence of hemorrhagic transformation (HT) groups.

Variables	No HT (*n* = 633)	HT (*n* = 117)	*P*
Age, years (IQR)	61 (53–68)	63 (55–70)	0.248
Male, *n* (%)	453 (71.6)	90 (76.9)	0.234
Cigarette smoking, *n* (%)	334 (52.8)	54 (46.2)	0.189
Alcohol consumption, *n* (%)	261 (41.2)	53 (45.3)	0.413
Hypertension, *n* (%)	315 (49.8)	69 (59.0)	0.067
Diabetes mellitus, *n* (%)	113 (17.9)	26 (22.2)	0.264
Coronary heart disease, *n* (%)	124 (19.6)	22 (18.8)	0.844
Atrial fibrillation, *n* (%)	54 (8.5)	20 (17.1)	0.004
Previous stroke, *n* (%)	84 (13.3)	19 (16.2)	0.391
Dyslipidemia, *n* (%)	262 (41.4)	51 (43.6)	0.658
Antihypertensive therapy, *n* (%)	246 (38.9)	54 (46.2)	0.139
Hypoglycemic therapy, *n* (%)	99 (15.6)	22 (18.8)	0.393
Antiplatelet therapy, *n* (%)	76 (12.0)	22 (18.8)	0.045
Baseline SBP, mmHg (IQR)	153 (138–165)	157 (145–169)	0.048
Baseline DBP, mmHg (IQR)	90 (81–98)	89 (81–97)	0.683
Baseline blood glucose, mmol/L (IQR)	7.5 (6.5–9.0)	8.3 (6.9–10.2)	0.007
Baseline NIHSS score (IQR)	8 (5–12)	10 (5–13)	0.042
ONT, min (IQR)	184 (140–230)	184 (150–225)	0.454
TOAST			0.005
Large-artery atherosclerosis, *n* (%)	225 (35.5)	49 (41.9)	
Small-vessel occlusion, *n* (%)	254 (40.1)	26 (22.2)	
Cardioembolism, *n* (%)	66 (10.4)	18 (15.4)	
Other determined etiology, *n* (%)	10 (1.6)	2 (1.7)	
Undetermined etiology, *n* (%)	78 (12.3)	22 (18.8)	
ALP, U/L (IQR)	75.1 (62.2–91.3)	76.3 (62.5–97.6)	0.617
Globulin, g/L (IQR)	27.0 (24.2–29.7)	27.7 (25.3–30.3)	0.022

HT, hemorrhagic transformation; IQR, inter quartile range; SBP, systolic blood pressure; DBP, diastolic blood pressure; NIHSS, National Institutes of Health Stroke Scale; ONT, onset to needle time; TOAST, Trial of Org 10172 in acute stroke treatment; ALP, alkaline phosphatase.

In the univariate analysis, globulin levels were associated with HT after IVT; while in the multivariate analysis, globulin levels were independently associated with hemorrhagic transformation, adjusted for Model 1 (OR = 1.058; 95% CI: 1.011–1.107; *P* = 0.014), Model 2 (OR = 1.052; 95% CI: 1.003–1.103; *P* = 0.035), Model 3 (OR = 1.055; 95% CI: 1.006–1.106; *P* = 0.026), and Model 4 (OR = 1.055; 95% CI: 1.006–1.107; *P* = 0.028). However, ALP level was not associated with HT in the univariate or multivariate analyses. The results of the univariate and multivariate logistic regression analyses for HT are shown in Table 3.

**TABLE 3:** Logistic regression analysis of the relationship between alkaline phosphatase (ALP) and globulin with hemorrhagic transformation (HT).

	Variables	OR	95% CI	*P*
Unadjusted	ALP	1.002	0.995–1.010	0.551
	Globulin	1.053	1.008–1.101	0.020
Model 1	ALP	1.003	0.996–1.010	0.446
	Globulin	1.058	1.011–1.107	0.014
Model 2	ALP	1.003	0.996–1.010	0.425
	Globulin	1.052	1.003–1.103	0.035
Model 3	ALP	1.003	0.996–1.011	0.378
	Globulin	1.055	1.006–1.106	0.026
Model 4	ALP	1.003	0.995–1.011	0.456
	Globulin	1.055	1.006–1.107	0.028

Model 1 was adjusted for age, sex; Model 2 was adjusted for Model 1 + vascular risk factors, including cigarette smoking, alcohol consumption, atrial fibrillation, coronary heart disease, hypertension, diabetes mellitus, dyslipidemia, and previous stroke; Model 3 was adjusted for Model 2 + history of previous medications, including antihypertensive therapy, hypoglycemic therapy, and antiplatelet therapy; Model 4 was adjusted for Model 3 + baseline NIHSS score, baseline SBP and DBP, baseline blood glucose, ONT, and TOAST.

*HT, hemorrhagic transformation; OR, odds ratio; 95% CI, 95% confidence interval; ALP, alkaline phosphatase; SBP, systolic blood pressure; DBP, diastolic blood pressure; NIHSS, National Institutes of Health Stroke Scale; ONT, onset to needle time; TOAST, Trial of Org 10172 in acute stroke treatment.*

### The association of alkaline phosphatase and globulin levels with poor outcome

We divided all eligible patients into poor outcome and favorable outcome groups. Comparisons of antihypertensive therapy, TOAST, baseline SBP, blood glucose, and NIHSS score were statistically significant (*P* < 0.05). ALP (71.4 vs. 76.6, *P* = 0.002) and globulin levels (26.6 vs. 27.4, *P* = 0.002) were significantly higher in patients with poor outcomes. Comparisons of the clinical characteristics according to favorable and poor outcomes are presented in Table 4. A comparison of the distribution of ALP and globulin levels in patients with poor outcomes is presented in Figure 2.

**TABLE 4:** Clinical characteristics of patients in favorable outcome and poor outcome.

Variables	Favorable outcome (*n* = 298)	Poor outcome (*n* = 452)	*P*
Age, years (IQR)	61 (52–68)	62 (54–69)	0.070
Male, *n* (%)	225 (75.5)	318 (70.4)	0.123
Cigarette smoking, *n* (%)	166 (55.7)	222 (49.1)	0.077
Alcohol consumption, *n* (%)	129 (43.3)	185 (58.9)	0.522
Hypertension, *n* (%)	143 (48)	241 (53.3)	0.153
Diabetes mellitus, *n* (%)	52 (17.4)	87 (19.2)	0.535
Coronary heart disease, *n* (%)	57 (19.1)	89 (19.7)	0.849
Atrial fibrillation, *n* (%)	30 (10.1)	44 (9.7)	0.881
Previous stroke, *n* (%)	36 (12.1)	67 (14.8)	0.286
Dyslipidemia, *n* (%)	129 (43.3)	184 (40.7)	0.483
Antihypertensive therapy, *n* (%)	102 (34.2)	198 (43.8)	0.009
Hypoglycemic therapy, *n* (%)	42 (14.1)	79 (17.5)	0.218
Antiplatelet therapy, *n* (%)	37 (12.4)	61 (13.5)	0.668
Baseline SBP, mmHg (IQR)	152 (137–163.3)	155 (140.3–168.8)	0.016
Baseline DBP, mmHg (IQR)	89 (80–97)	90 (82–98)	0.249
Baseline blood glucose, mmol/L (IQR)	7.4 (6.4–8.8)	7.7 (6.7–9.4)	0.003
Baseline NIHSS score (IQR)	6 (4–10)	10 (6–13)	<0.001
ONT, min (IQR)	180.5 (137.5–230)	184.5 (146.3–228.8)	0.662
TOAST			<0.001
Large-artery atherosclerosis, *n* (%)	75 (25.2)	199 (44.0)	
Small-vessel occlusion, *n* (%)	144 (48.3)	136 (30.1)	
Cardioembolism, *n* (%)	33 (11.1)	51 (11.3)	
Other determined etiology, *n* (%)	9 (3)	3 (0.7)	
Undetermined etiology, *n* (%)	37 (12.4)	63 (13.9)	
ALP, U/L (IQR)	71.4 (60.1–87.2)	76.6 (63.5–94.3)	0.002
Globulin, g/L (IQR)	26.6 (23.9–29.3)	27.4 (24.7–30.4)	0.002

IQR, inter quartile range; SBP, systolic blood pressure; DBP, diastolic blood pressure; NIHSS, National Institutes of Health Stroke Scale; ONT, onset to needle time; TOAST, Trial of Org 10172 in acute stroke treatment; ALP, alkaline phosphatase.

Logistic regression analyses were used to explore the association between ALP and globulin levels and poor outcome. In multivariate analysis, ALP was independently associated with poor outcome adjusted by Model 1 (OR = 1.010; 95% CI: 1.003–1.016; *P* = 0.003), Model 2 (OR = 1.010; 95% CI: 1.004–1.016; *P* = 0.002), Model 3 (OR = 1.011; 95% CI: 1.005–1.018; *P* = 0.001), and Model 4 (OR = 1.009; 95% CI: 1.002–1.016; *P* = 0.010). Globulin level adjusted by Model 1 (OR = 1.055; 95% CI: 1.018–1.094; *P* = 0.003), Model 2 (OR = 1.059; 95% CI: 1.020–1.099; *P* = 0.003), Model 3 (OR = 1.056; 95% CI: 1.017–1.096; *P* = 0.004) and Model 4 (OR = 1.062; 95% CI: 1.020–1.107; *P* = 0.004) was independently associated with poor outcome. Table 5 shows the results of the logistic regression analysis for poor outcomes after IVT.

**TABLE 5:** Logistic regression analysis of the relationship between alkaline phosphatase (ALP) and globulin with poor outcome.

	Variables	OR	95% CI	*P*
Unadjusted	ALP	1.010	1.004–1.016	0.001
	Globulin	1.063	1.026–1.101	0.001
Model 1	ALP	1.010	1.003–1.016	0.003
	Globulin	1.055	1.018–1.094	0.003
Model 2	ALP	1.010	1.004–1.016	0.002
	Globulin	1.059	1.020–1.099	0.003
Model 3	ALP	1.011	1.005–1.018	0.001
	Globulin	1.056	1.017–1.096	0.004
Model 4	ALP	1.009	1.002–1.016	0.010
	Globulin	1.062	1.020–1.107	0.004

Model 1 was adjusted for age, sex; Model 2 was adjusted for Model 1 + vascular risk factors, including cigarette smoking, alcohol consumption, atrial fibrillation, coronary heart disease, hypertension, diabetes mellitus, dyslipidemia, and previous stroke; Model 3 was adjusted for Model 2 + history of previous medications, including antihypertensive therapy, hypoglycemic therapy, and antiplatelet therapy; Model 4 was adjusted for Model 3 + baseline NIHSS score, baseline SBP and DBP, baseline blood glucose, ONT, and TOAST.

OR, odds ratio; 95% CI, 95% confidence interval; ALP, alkaline phosphatase; SBP, systolic blood pressure; DBP, diastolic blood pressure; NIHSS, National Institutes of Health Stroke Scale; ONT, onset to needle time; TOAST, Trial of Org 10172 in acute stroke treatment.

### Individualized prediction model

**FIGURE 3:**
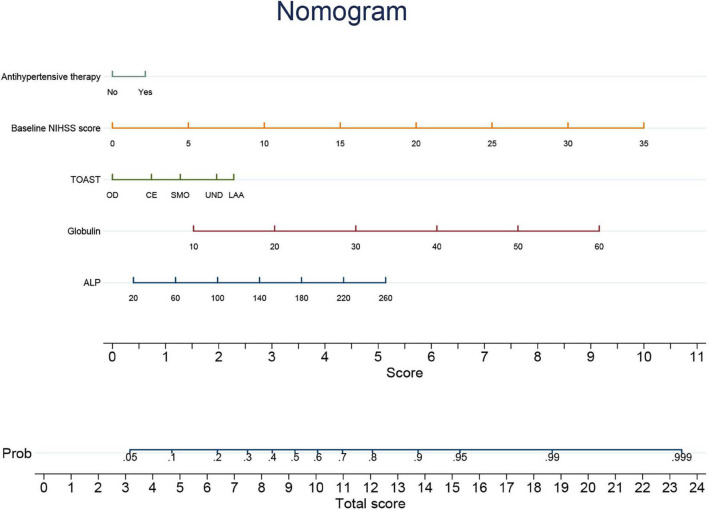
Nomogram of individualized prediction model to predict 3-month poor outcome. NIHSS, National Institutes of Health Stroke Scale; TOAST, Trial of Org 10172 in acute stroke treatment; LAA, large-artery atherosclerosis; SMO, small-vessel occlusion; CE, cardioembolism; OD, other determined etiology; UND, undetermined etiology; ALP, alkaline phosphatase.

**FIGURE 4:**
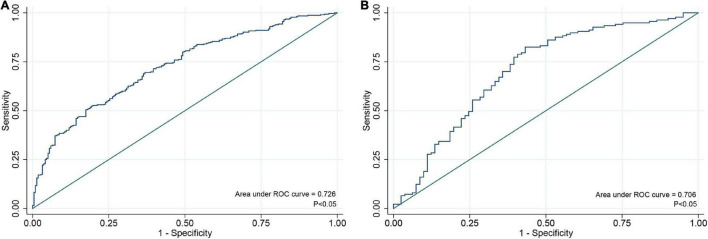
ROC curve for individualized prediction model to predict 3-month poor outcome in training set **(A)** and validation set **(B)**. ROC, receiver operating characteristic.

**FIGURE 5:**
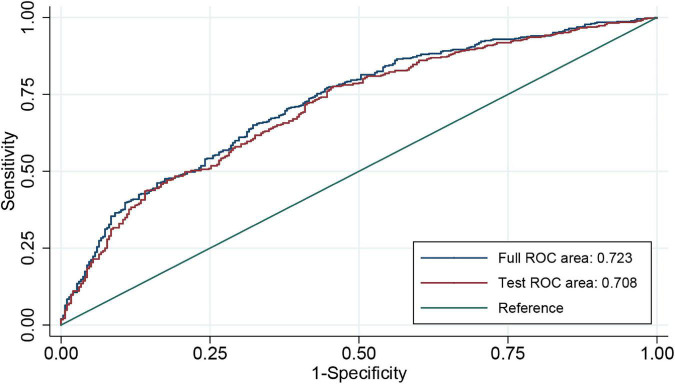
ROC curve for 10-fold cross-validation. ROC, receiver operating characteristic.

An individualized prediction model for functional outcomes was established based on the results of univariate analysis. The prediction model was composed of serum ALP level, globulin level, baseline NIHSS score, TOAST score, and history of antihypertensive therapy. A nomogram was developed, as shown in Figure 3. The discriminative performance of the model was evaluated using the area under the curve (AUC) of the training set (AUC, 0.726; 95% CI: 0.683–0.768). The model was tested using the AUC of the validation set (AUC, 0.706; 95% CI: 0.631–0.781). After a 10-fold cross-validation was performed on all training and validation sets data, the full ROC area was 0.723, and the test ROC area was 0.708, indicating that the present model was stable. Figure 4 shows the ROC curve for the training and validation sets, and the ROC curve for the 10-fold cross-validation is shown in Figure 5. The *P*-value for the Hosmer-Lemeshow test in the training set was 0.978 > 0.05, and in the validation set was 0.148 > 0.05, suggesting good calibration.

## Discussion

This study found that ALP level was an independent risk factor for a poor outcome but not an independent risk factor for HT after thrombolysis. The globulin level was an independent risk factor for a poor outcome and HT after thrombolysis. The results were statistically significant in all four models after adjusting for confounders. Our results indicate that serum ALP and globulin levels might be reliable predictors of the prognosis of patients with AIS who have undergone IVT treatment.

The association between ALP levels and clinical outcomes of stroke has been reported in previous studies. Ryu et al. (2010) found that increased serum ALP levels o study found that ALP level was an inculd independently predict all-cause and vascular death after ischemic or hemorrhagic stroke. Zong et al. (2018) indicated that ALP was an independent predictor of poor long-term functional outcomes after stroke. However, the relationship between ALP levels and clinical outcomes remains unclear in patients undergoing IVT. In this context, our study further presented novel findings that serum ALP level could also be regarded as a predictor of a 3-month poor prognosis in IVT-treated patients with AIS. The probable mechanism might be that elevated ALP levels were associated with increased vascular calcification (Kim et al., 2013; Haarhaus et al., 2017). ALP is a key regulator of the phosphate/pyrophosphate ratio (Azpiazu et al., 2019), which plays a role in vascular calcification (Lomashvili et al., 2014). Intracranial vascular calcification has been shown to be a predictor of poor outcomes in patients undergoing IVT (Yu et al., 2021). In addition, another potential mechanism was that ALP was also considered a marker of inflammation, malnutrition, and metabolic syndrome, which might lead to adverse prognosis in patients with stroke (Tonelli et al., 2009; Kim et al., 2013; Wannamethee et al., 2013).

Only a few studies have currently reported an association between ALP levels and HT. Liu et al. (2016) showed that ALP could not predict HT in patients with cardioembolic stroke. Their findings are consistent with the present study’s results in patients with all stroke subtypes who received IVT, indicating that ALP may not be regarded as an indicator of HT in patients with stroke.

Another major finding of our study was the association between elevated globulin levels and clinical outcomes in patients with AIS after IVT. Previous studies have illustrated that the globulin-to-prealbumin ratio predicted the 3-month functional outcome in patients with AIS receiving rt-PA therapy (Li et al., 2022); however, they did not demonstrate the predictive power of globulin level as a single indicator. To the best of our knowledge, our study is the first to confirm that globulin level is an independent predictor of poor prognosis in patients with AIS after IVT. Association between globulin and HT in patients undergoing IVT. An increase in globulin level is an independent risk factor for HT has been illustrated in patients receiving intra-arterial thrombolysis (Xing et al., 2014).

Moreover, Zhong et al. (2021) recently reported that the globulin level was also a risk factor for HT in patients with AIS after IVT. However, they measured globulin levels before thrombolysis. The utility of rt-PA may induce inflammatory responses (Miyazaki et al., 2011; Yang et al., 2019) and may impact changes in globulin levels; hence, it seems to be more rational to consider globulin tested after IVT as a predictor of clinical outcome. Nevertheless, whether post-IVT globulin post IVT could predict HT remains unclear. The present study demonstrated an independent association between the globulin levels tested after IVT and HT in a larger cohort, adding an important supplement to the current literature.

Inflammation was one of the possible mechanisms of the relationship between globulin and poor prognosis (Lasek-Bal et al., 2019; Montellano et al., 2021). Globulin is a protein produced by immune organs and reflects the severity of inflammation (Ballow, 1997). Inflammatory cytokines, such as interleukin 6, interleukin 1, and tumor necrosis factor α, are expressed in the liver through acute-phase protein genes. They induce an increase in acute phase response proteins, including globulin (Gabay and Kushner, 1999). Increased inflammatory cytokine levels in patients with acute ischemic stroke are associated with a poor prognosis (Um et al., 2005). Furthermore, inflammatory cytokines can upregulate matrix metalloproteinase-9 (Huwiler et al., 2003), which is an independent biochemical predictor of HT in stroke patients (Castellanos et al., 2003).

As mentioned previously, serum ALP and globulin levels were found to be risk factors for a poor prognosis, and testing of these biomarkers is easy and widely used in clinical practice. We further constructed a nomogram for predicting functional outcomes at 3 months based on serum ALP and globulin levels, baseline NIHSS score, TOAST, and history of antihypertensive therapy. The individualized prediction model displayed by the nomogram can predict the probability of a 3-month poor outcome in individual patients. Our results showed that the model had good discrimination and calibration in the training and validation sets for individualized prediction of the 3-month poor outcome.

This study has some limitations. First, this study retrospectively analyzed data from a single medical center. Second, blood samples were only tested after intravenous thrombolytic therapy, and changes that may be affected by IVT treatment were not observed. In addition, the individualized prediction model has not been validated in other populations; therefore, the predictions for other populations need further validation. Larger prospective cohorts should be established to observe the predictive roles of ALP and globulin in patients with AIS who receive IVT and to explore the underlying mechanisms.

## Conclusion

We found that higher serum ALP and globulin levels were independently associated with a poor outcome in patients with AIS treated with IVT. Additionally, we found that higher serum globulin levels were independently associated with HT after IVT. An individualized prediction model based on serum ALP and globulin levels to predict the 3-month poor outcome was constructed and demonstrated good discrimination and calibration. The findings of this study could help clinicians predict outcomes and provide new therapeutic targets for improving the prognosis of patients with AIS undergoing IVT.

## Data availability statement

The raw data supporting the conclusions of this article will be made available by the authors, without undue reservation.

## Ethics statement

The studies involving human participants were reviewed and approved by the Ethics Review Committee of the First Hospital of Jilin University. The patients/participants provided their written informed consent to participate in this study.

## Author contributions

H-JZ, XS, B-FX, and YY were responsible for conceptualization. H-JZ, XS, Z-NG, and YQ performed data collection and analysis. H-JZ and XS drafted the manuscript. HJ, Y-YS, and M-QW helped to revise the manuscript. All authors contributed to the article and approved the submitted version.

## Conflict of interest

The authors declare that the research was conducted in the absence of any commercial or financial relationships that could be construed as a potential conflict of interest.

## Publisher’s note

All claims expressed in this article are solely those of the authors and do not necessarily represent those of their affiliated organizations, or those of the publisher, the editors and the reviewers. Any product that may be evaluated in this article, or claim that may be made by its manufacturer, is not guaranteed or endorsed by the publisher.
